# Renal Water Molecular Diffusion Characteristics in Healthy Native Kidneys: Assessment with Diffusion Tensor MR Imaging

**DOI:** 10.1371/journal.pone.0113469

**Published:** 2014-12-03

**Authors:** Zhenfeng Zheng, Huilan Shi, Jing Zhang, Yunting Zhang

**Affiliations:** 1 Department of Nephrology, General Hospital of Tianjin Medical University, Heping District, Tianjin, China; 2 Department of Radiology, General Hospital of Tianjin Medical University, Heping District, Tianjin, China; University Medical Center Utrecht, Netherlands

## Abstract

**Background:**

To explore the characteristics of diffusion tensor imaging (DTI) and magnetic resonance (MR) imaging in healthy native kidneys.

**Methods:**

Seventy-three patients without chronic kidney disease underwent DTI-MRI with spin echo-echo planar (SE-EPI) sequences accompanied by an array spatial sensitivity encoding technique (ASSET). Cortical and medullary mean, axial and radial diffusivity (MD, AD and RD), fractional anisotropy (FA) and primary, secondary and tertiary eigenvalues (λ_1_, λ_2_, λ_3_) were analysed in both kidneys and in different genders.

**Results:**

Cortical MD, λ_2_, λ_3_, and RD values were higher than corresponding medullary values. The cortical FA value was lower than the medullary FA value. Medullary λ_1_ and RD values in the left kidney were lower than in the right kidney. Medullary λ_2_, and λ_3_ values in women were higher than those in men. Medullary FA values in women were lower than those in men. Medullary FA (*r* = 0.351, *P* = 0.002) and λ_1_ (*r* = 0.277, *P* = 0.018) positively correlated with eGFR. Medullary FA (*r* = −0.25, *P* = 0.033) negatively correlated with age.

**Conclusions:**

Renal water molecular diffusion differences exist in human kidneys and genders. Age and eGFR correlate with medullary FA and primary eigenvalue.

## Background

A major function of the kidney is to maintain water balance and solute concentration. The physiological function of the glomeruli and tubules are filtration and reabsorption of water and solutes. Understanding water diffusion in the kidney will help better manage many kidney diseases [1]. Laboratory parameters, such as serum creatinine or estimated glomerular filtration rate (eGFR), are commonly used for providing renal filtration information. However, these parameters are not very sensitive or specific. They do not provide split renal function or renal tubule reabsorption information. Renal biopsy is often performed to assess renal pathological damage. However, this invasive procedure can have serious complications and is restricted in its clinical application.

Diffusion tensor imaging (DTI) provides insight into the structural properties of tissue. Diffusion imaging has been extensively applied to the brain, and as a result, is increasingly being used in clinical environments. Renal diffusion imaging is limited by the same problems magnetic resonance (MR) imaging faced when first introduced, namely motion artifacts caused by arterial pulsations and respiratory motion. Breath-hold imaging [2]–[4] and respiratory triggering [5] have been suggested as techniques to overcome these problems. DTI is a development from diffusion-weighted MRI, which allows the quantification of diffusion in different directions. Diffusion anisotropy is related to structural organization and therefore could be compromised in a pathological process. Molecular diffusion, however, is a three-dimensional process which can occur with different probabilities in each direction, i.e. in an anisotropic manner [2]. This is the case in the kidney, which has a well-defined structure with tubules, collecting ducts and vessels radially oriented towards the pelvis and in which molecules move in a preferential direction [1], [6]. The measurement of global diffusion and the direction of the diffusion is necessary to investigate molecular diffusion in the kidney [1]. DTI can provide three indices related to the magnitude of diffusivity from the mathematical description of the system. These indices are the mean, axial and radial diffusivity (MD, AD, and RD, respectively) [7]. MD is the average of the average diffusion coefficient (ADC) in all three (*x*, *y*, and *z*) directions and is a reflection of the magnitude of the tensor that describes the system. AD is the first eigenvalue, and occurs in the longitudinal direction of the tensor. RD is the average of the second and third eigenvalues. It is related to diffusion along the radial direction. The three indices are related to diffusional anisotropy and fractional anisotropy (FA) [8].

The measurement of MD, AD, RD and FA in a healthy kidney has not yet been reported in the literature. The aim of our study was to evaluate the diffusivity characteristics of the renal cortex and medulla and provide baseline data for future studies.

## Materials and Methods

### Study protocol

The study was designed as an observational, open study. Between July 2013 and September 2013, seventy-three patients who underwent abdominal MR imaging with a 3.0T machine were evaluated. Study protocol was in compliance with the Helsinki Declaration. This study was approved by General Hospital of Tianjin Medical University Ethic’s committees. All participants gave their written informed consent prior to study participation. Inclusion criteria included: 1) No abnormalities of urinary analysis, blood tests or medical imaging within the last three months. 2) eGFR greater than 60 ml/min/1.73 m^2^ in the last three months. 3) No use of angiotensin converting enzyme inhibitors (ACEI), angiotensin receptor blockers (ARB), calcium channel blockers (CCB), diuretics, or vasodilators in the last two weeks, as these agents could impact renal blood fluid and renal oxygen consumption. Physical examination, including body height and weight, blood pressure, and heart rate, and serum creatinine, and urine tests were completed in all participants. BMI, BSA and eGFR were calculated. Serum creatinine levels were considered as current, if they were obtained within 14 days of MR imaging. The current eGFR was calculated using the CKD-EPI formula and the current serum creatinine value [9].

### Magnetic Resonance Imaging Techniques

Magnetic resonance imaging was performed using a 3.0-T Imager (GE Healthcare, Milwaukee, Wisconsin, U.S.). The scanner had a maximum gradient strength of 50 mT/m and a slew rate of 200 mT/m/sec. A Torsopa eight-channel body coil was also used. Morphologic images were acquired using T1 weighted fat-suppressed technique. Images were acquired using T_1_INPHASE+FAT sequence. The field of view (FOV) was 380×380 mm section thickness 7.0 mm, section width 1.0 mm, and repetition time (TR)/echo time (TE) 180/2.1. DTI-MRI was performed using a SE-EPI sequence accompanied by ASSET. Acquisition parameters were TR/TE 2000/94.5, FOV 380×380 mm, phase fov 1.0, section thickness 7.0 mm, section width 1.0 mm, matrix size 128×128, NEX 2, diffusion weighted coefficient *b* value 800 s/mm^2^, diffusion sensitivity gradient direction 6, acquiescent section number 5–7, and scan time 18 sec. br2eath hold, 15 sec; bandwidth, 19.23 kHz; acquiescent plane, coronal; diffusion sensitive gradient direction, 6; six sensitive gradient directions could express as an icosahedron with 20 faces and 12 vertices. The unit vectors for the icosahedric acquisition may be taken as follows: *g*_1_ = (c_1_, c_2_, 0); *g*_2_ = (c_1_,−c_2_, 0); *g*_3_ = (0, c_1_, c_2_); *g*_4_ = (0, c_1_,–c_2_); *g*_5_ = (c_2_, 0, c_1_); *g*_6_ = (−c_2_, 0, c_1_). 
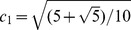
; 
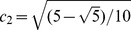
 [10]. Data acquired in each of three breath holds included two *b* values (*b* = 0 and 800 sec/mm^2^). The post-processing tools of the DTI and ROIs is AW FuncTool (GE Medical Systems LLC, 3200N. Grandview Boulevard Waukesha, WI 53188, USA).

### DTI Analysis

An average diffusion coefficient along each direction was derived from the DW images (*b* = 0 and 800 sec/mm^2^), as follows [11]:
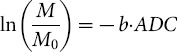



The eigenvectors (ν_1_, ν_2_, ν_3_) and eigenvalues (λ_1_, λ_2_, λ_3_) of the diffusion tensor were determined. The DTI eigenvalues λ_i_ were calculated and used to derive the mean parametric maps for the MD, FA, direction encoded color maps, principal diffusivities (λ_1_, λ_2_, λ_3_), and primary diffusion eigenvector. The primary eigenvalue λ_1_ (axial or longitudinal diffusivity) was the largest and least restricted diffusivity. In a medullary tubule this would reflect motion in the tubule. The direction of the tubule was specified by the primary diffusion eigenvector ν_1_. The secondary and tertiary eigenvalues (λ_2_, λ_3_) (and their average) reflect lower restricted diffusion orthogonal to ν_1_ (radial or transverse diffusion). In the medulla this corresponds to cross-tubule or transtubular flow [6]. Three indices related to the magnitude of diffusivity, MD, AD, and RD, were derived from the tensor [7]. MD was the averages of the ADC along all directions, a reflection of the magnitude of the tensor:
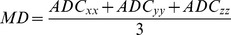



AD was the first eigenvalue, corresponding to the longitudinal direction of the tensor:
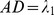



RD was the average of the second and third eigenvalues. This corresponds to diffusion in the radial direction:
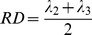



The index related to diffusional anisotropy was FA [8]. FA quantified the difference of directionally dependent diffusion within a voxel of interest. In a cylindrical model, FA is 1. FA is 0 if the tensor is spherical. FA parametric maps were calculated to depict the degree of diffusion anisotropy:
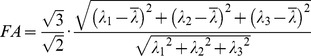
With



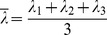



Ellipsoid regions of interest (ROIs) covering at least 10 pixels each were drawn on the anatomic templates. Large ROIs were placed in the upper pole, middle pole, and lower pole of the cortex and medulla. Single total ROIs were created separately for the cortex and the medulla by averaging individual ROIs, yielding two ROIs for each subsection. ROIs in the right and left kidneys were averaged for each subject separately for the cortex and the medulla, after excluding significant right-left differences. The reader was blinded to patient clinical information. The order of patients was randomized.

### Statistical Analysis

All of the quantitative measurements were expressed as the mean±standard deviation. In order to facilitate further analysis, cortical and medullary DTI parameters were averaged in those patients with two kidneys. A Kolmogorov-Smirnov test was used to test for normal distribution. T-test analyses were performed to detect statistically significant differences when the data revealed a normal distribution. A general linear model for factorial design variance analysis was used with the Bonferroni method for multiple comparisons. Principal component and factorial analysis were used to detect independent variable colinearity and extract potential factors according to eigenvalues. Two-sided *P* values <0.05 were regarded as statistically significant. Statistical analyses were performed using SPSS software package version 17.0.

## Results

### Demographic and anthropometric characteristics

**Figure 1:**
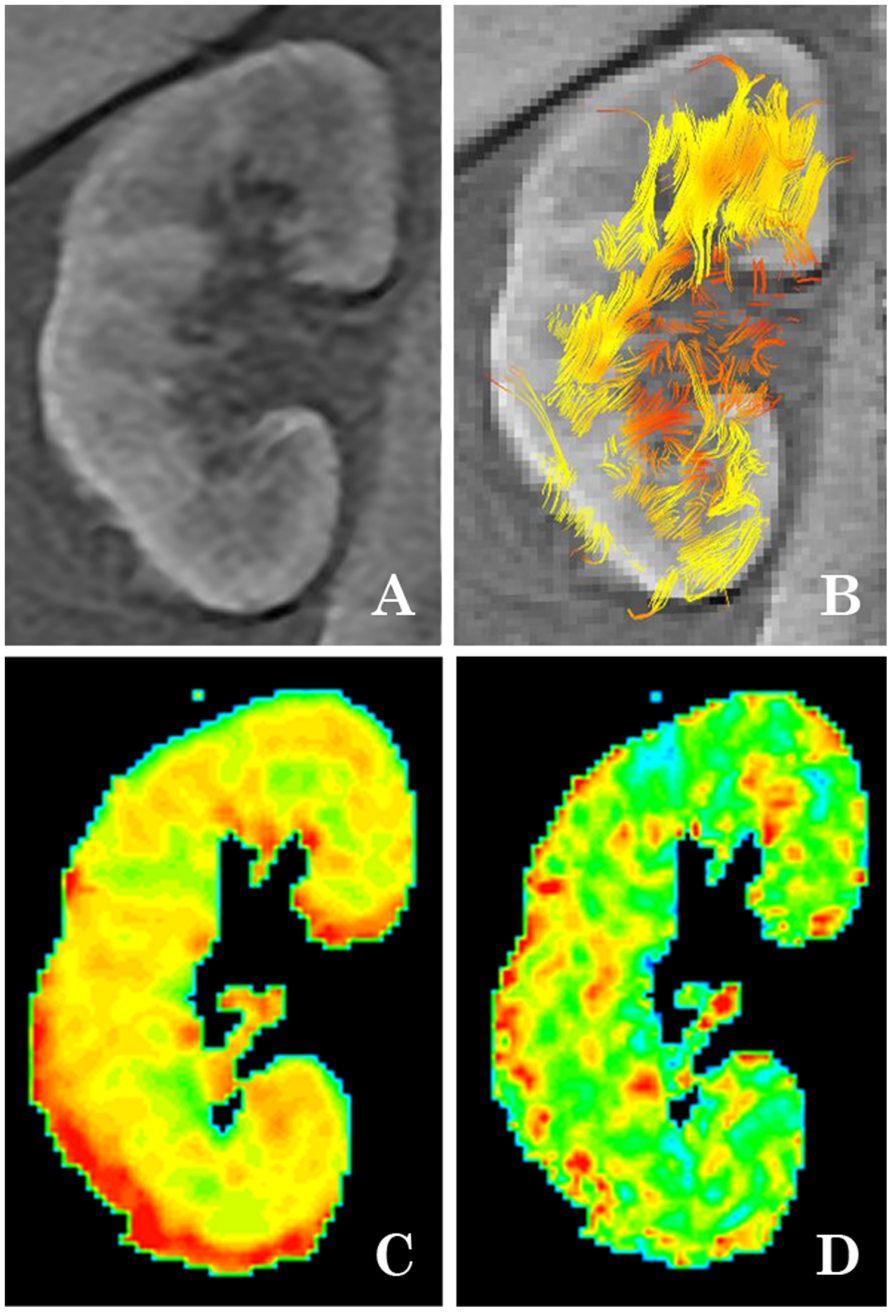
Anatomic and DTI-MRI map of native healthy kidneys. (A) T_1_INPHASE fat-suppression background reference map; (B) DTI tractographic map; (C) DTI MD pseudo-color map; (D) DTI FA pseudo-color map.

Seventy-three subjects, including sixty-three patients and ten healthy volunteers, underwent DTI MRI imaging. The detailed demographic and anthropometric characteristics are summarized in Table 1. A fat-suppressed background reference map, DTI pseudo-color map and DTI tractographic map are shown in Figure 1.

**Table 1:** Patient demographics and anthropometric indices.

Item	Cortex	Medulla	*P* value
MD(µm^2^/ms)	2.23±0.14	2.06±0.14	<0.0001
FA	0.24±0.05	0.32±0.05	<0.0001
λ_1_(µm^2^/ms)	6.29±0.21	6.28±0.26	0.81
λ_2_(µm^2^/ms)	5.67±0.15	5.43±0.15	<0.0001
λ_3_(µm^2^/ms)	5.24±0.12	4.96±0.13	<0.0001
RD(µm^2^/ms)	5.46±0.12	5.19±0.13	<0.0001

### Impact of gender, renal location, and kidney side on DTI parameters

**Figure 2:**
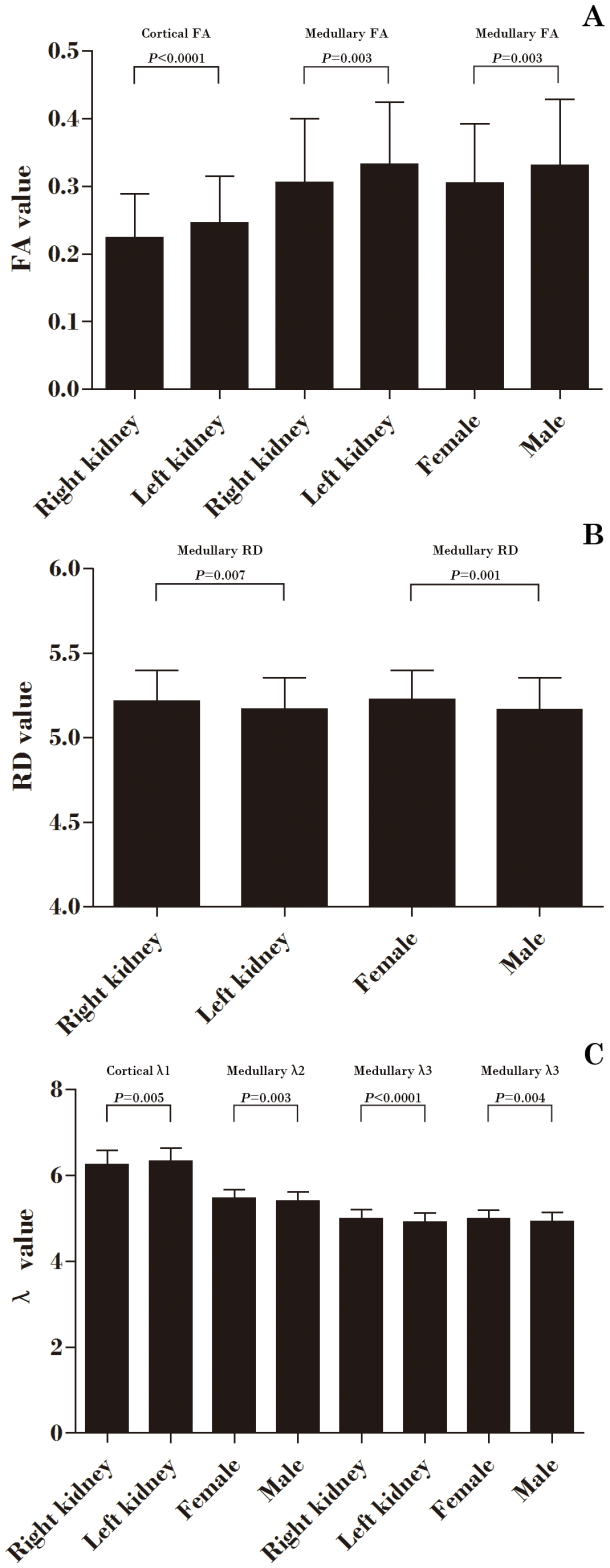
Cortical and medullary DTI parameters by kidney and gender. (A) Comparison of cortical and medullary FA values by kidney and gender; (B) Comparison of medullary RD values by kidney and gender; (C) Comparison of cortical and medullary λ values by kidney and gender.

Cortical MD, λ_2_, λ_3_, and RD values were higher than corresponding medullary values. There was no difference in cortical and medullary λ_1_ value. Cortical FA value was lower than medullary FA value (Table 2). Cortical FA, λ_1_ value and medullary FA in the left kidney were higher than similar values in the right kidney. Medullary λ_1_ and RD values in the left kidney were lower than similar values in the right kidney. Medullary λ_2_ and λ_3_ values in women were higher than similar values in men. Medullary FA values in women were lower than those in men (Table 3 and Table 4, Figure 2).

**Table 2:** DTI parameters in the cortex and medulla.

Item	Right kidney	Left kidney	*P* value
Cortical MD (µm^2^/ms)	2.23±0.20	2.24±0.17	0.42
Medullary MD (µm^2^/ms)	2.07±0.18	2.05±0.19	0.34
Cortical FA	0.22±0.07	0.25±0.07	<0.0001
Medullary FA	0.31±0.09	0.33±0.09	0.003
Cortical λ_1_ (µm^2^/ms)	6.25±0.33	6.33±0.30	0.005
Medullary λ_1_ (µm^2^/ms)	5.44±0.23	5.42±0.21	0.18
Cortical λ_2_ (µm^2^/ms)	5.86±0.23	5.66±0.20	0.28
Medullary λ_2_ (µm^2^/ms)	5.43±0.23	5.42±0.21	0.36
Cortical λ_3_ (µm^2^/ms)	5.25±0.16	5.22±0.18	0.11
Medullary λ_3_ (µm^2^/ms)	4.99±0.21	4.92±0.21	<0.0001
Cortical RD (µm^2^/ms)	5.47±0.17	5.44±0.16	0.12
Medullary RD (µm^2^/ms)	5.21±0.18	5.17±0.19	0.007

**Table 3:** DTI parameter index value by kidney.

Item	Female	Male	*P* value
Cortical MD (µm^2^/ms)	2.25±0.18	2.22±0.18	0.23
Medullary MD (µm^2^/ms)	2.07±0.17	2.05±0.20	0.24
Cortical FA	0.23±0.07	0.24±0.07	0.77
Medullary FA	0.31±0.09	0.33±0.10	0.003
Cortical λ_1_ (µm^2^/ms)	6.30±0.30	6.28±0.32	0.52
Medullary λ_1_ (µm^2^/ms)	6.25±0.33	6.31±0.40	0.06
Cortical λ_2_ (µm^2^/ms)	5.69±0.22	5.66±0.21	0.11
Medullary λ_2_ (µm^2^/ms)	5.46±0.21	5.40±0.22	0.003
Cortical λ_3_ (µm^2^/ms)	5.24±0.16	5.23±0.18	0.37
Medullary λ_3_ (µm^2^/ms)	4.99±0.21	4.93±0.21	0.004
Cortical RD (µm^2^/ms)	5.47±0.16	5.44±0.17	0.12
Medullary RD (µm^2^/ms)	5.22±0.17	5.16±0.19	0.001

**Table 4:** DTI parameter index value by gender.

Indexes	KMO test	Bartlett’s test
Physiological items	0.602	<0.0001
DTI items	0.67	<0.0001

Footnote: KMO, Kaiser-Meyer-Olkin measure of sampling adequacy;

Bartlett’s, Bartlett’s test of sphericity.

physiological items including: age, gender, height, weight, BMI, BSA, eGFR.

DTI items including: MD, FA, λ_1_, λ_2_, λ_3_, RD.

### Impact of Demographic and anthropometric characteristics on DTI parameter

Factor analysis showed colinearity in both demographic and anthropometric indices and DTI parameter (Table 5). The effect of colinearity was to make the regression coefficients unreliable. We extracted three and four factors from demographic and anthropometric indices and DTI parameters, respectively. The accumulation of variance explained by extracted factors was 92.5% and 95.9%, respectively (Table 6 and Table 7). Formulas of factor scores are listed below.

**Table 5:** Factor analysis of physiological and DTI indices.

Component	Initial Eigenvalue	Proportion of variance (%)	Cumulation of variance (%)
Physiological items			
Factor 1	3.253	40.392	40.392
Factor 2	1.859	27.869	68.261
Factor 3	1.363	24.235	92.496
DTI items			
Factor 1	6.724	34.858	34.858
Factor 2	2.638	28.944	63.802
Factor 3	1.169	18.408	82.21
Factor 4	0.973	13.652	95.862

**Table 6:** Variance by principal component and rotational sum.

Factors	
Physiological items	
Factor 1	Weight, BMI, BSA
Factor 2	Gender, height
Factor 3	Age, eGFR
DTI items	
Factor 1	Cortical MD, cortical RD, cortical λ_1_, cortical λ_2_, cortical λ_3_
Factor 2	Medullary MD, medullary RD, medullary λ_2_, medullary λ_3_
Factor 3	Medullary FA, medullary λ_1_
Factor 4	Cortical FA

**Table 7:** Extracted factors from tested indexes.

Characteristic	Result
Age (years)	49.79±15.48
Gender (M/F)	39/34
Height (m)	1.68±0.07
Weight (kg)	66.21±13.24
BMI (kg/m^2^)	23.44±4.22
BSA (m^2^)	1.74±0.19
eGFR (ml/min/1.73 m^2^)	104.22±17.43
Comorbidity, n (%)	
Hypertension	21(28.77)
Diabetes	14(19.18)
Coronary artery disease	11(15.07)
Cerebrovascular disease	8(10.95)
Gastrointestinal disease	6(8.22)
Liver disease	5(6.85)
Thyroid disease	3(4.11)
Autoimmune disease	2(2.74)
Neoplasia	2(2.74)
Gynecologic inflammation	1(1.37)

Abbreviation: M, male; F, female; BMI, body mass index; BSA, body surface area; eGFR, estimated glomerular filtration.

**Figure 3:**
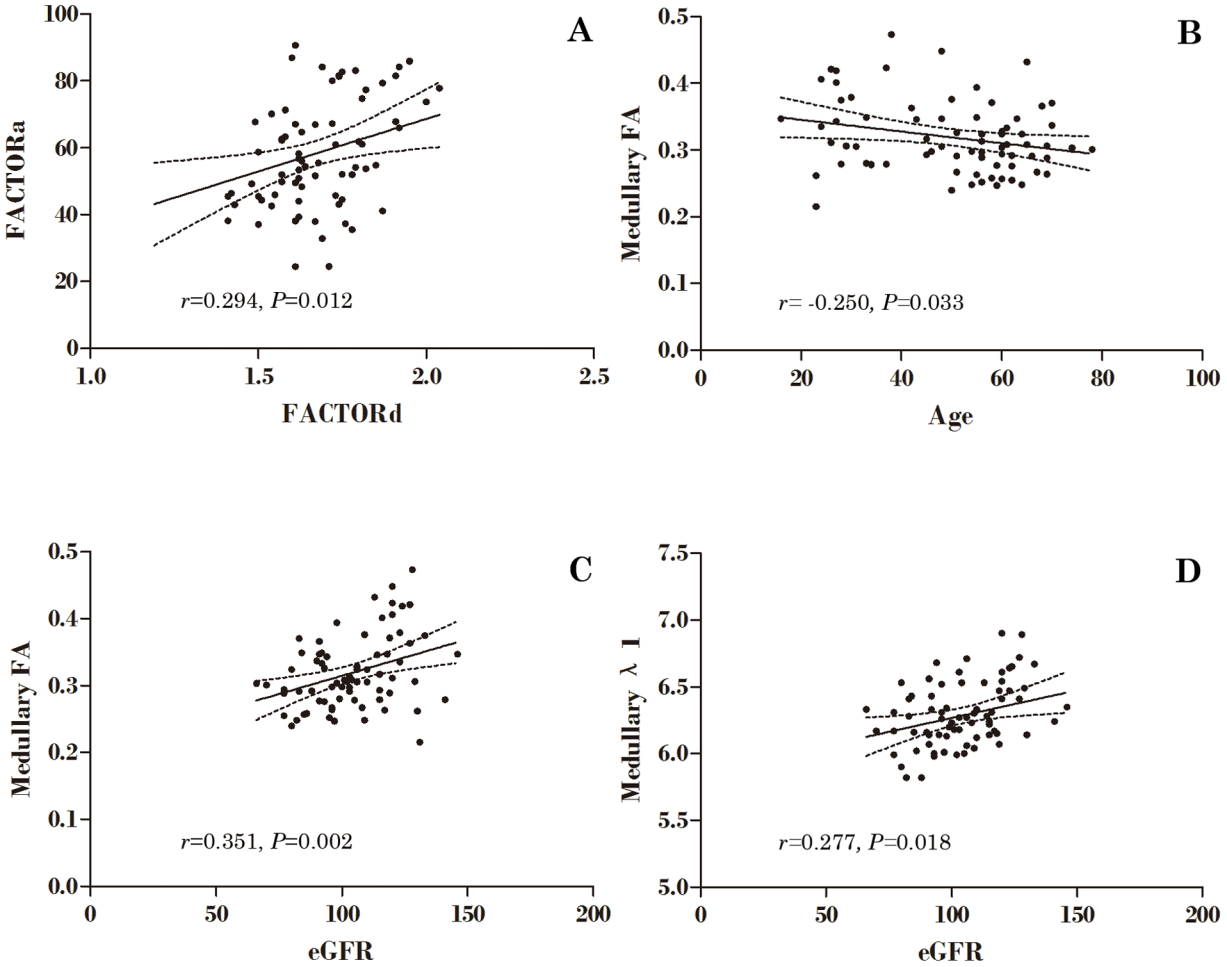
Correlation of renal cortical and medullary DTI parameters and anthropometric indices. (A) Anthropometric factor 3 versus DTI factor 3; (B) Age versus medullary FA value; (C) eGFR versus medullary FA value; (D) eGFR versus medullary λ_1_ value.

Factors from demographic & anthropometric indexes:



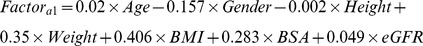


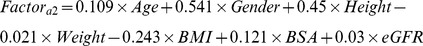


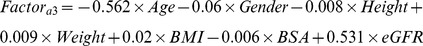
Factors from DTI parameters:



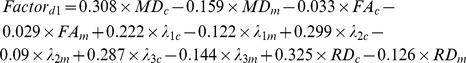


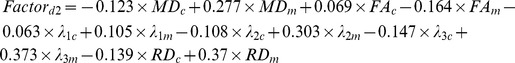


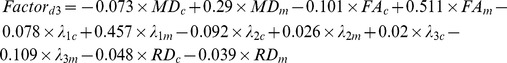


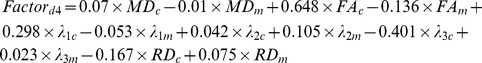
Annotations: MD_c_, cortical MD; MD_m_, medullary MD; FA_c_, cortical FA; FA_m_, medullary FA; λ_1c_, cortical λ_1_; λ_1m_, medullary λ_1_; λ_2c_, cortical λ_2_; λ_2m_, medullary λ_2_; λ_3c_, cortical λ_3_; λ_3m_, medullary λ_3_; RD_c_, cortical RD; RD_m_, medullary RD;

Correlation analysis showed that Factor_a3_ positively correlated with Factor_d3_ (*r* = 0.294, *P* = 0.012). Medullary FA and λ_1_ positively correlated with eGFR and medullary FA negatively correlated with age (Figure 3).

## Discussion

In this pilot study, we identified significantly higher mean cortical MD, λ_2_, λ_3_, and RD. The primary eigenvalue λ_1_ (axial or longitudinal diffusivity) was the largest and least restricted diffusivity and, in a medullary tubule, reflected motion along the length of the tubule. This included intratubular flow, whose direction was specified by the primary diffusion eigenvector, The secondary and tertiary eigenvalues (λ_2_, λ_3_) (and their average) reflected lower restricted diffusion orthogonal to λ_1_ (radial or transverse diffusion). In the medulla this corresponded to cross-tubule or transtubular motion [6]. These differences in DTI indices reflected the renal anatomic and physiological structure. The chief function of the kidney is filtration of plasma and formation of urine. The renal blood flow, in particular blood flow to the renal cortex, is much greater than that needed for the metabolic requirements of the kidney. Most blood is directed towards the renal cortex to optimize glomerular filtration and reabsorption of solute. The renal cortex requires rich perfusion to function properly, while the renal medulla requires limited blood flow. The maintenance of a relatively low medullary blood flow appears to be critical for maintaining the cortical-medullary solute gradient and, therefore, urinary concentrating mechanisms [12]. Blood is supplied to the renal medulla from the vasa recta capillaries. The vasa recta capillaries arise from the efferent arterioles of the juxtamedullary glomeruli, which comprise about 10% of all glomeruli in the kidney [12]. While all blood flow to the kidney enters the renal cortex, only about 10% reaches the renal medulla [12]. Blood flow in the outer and inner medulla is about 40% and 10%, respectively, of that in the renal cortex [12]. Kidneys filter the entire plasma volume every 30 min, reabsorbing two-thirds of the filtrate in the proximal tubule [13]. Because aquaporin-1 (AQP_1_) has been shown to be present both in the apical and basolateral membranes of the proximal tubular cells, transcellular water transport is thought to occur in both these directions [14]. In a study of aquanporin-1 knockout mice, the transepithelial water permeability of the proximal tubule was reduced 78% and the fluid in the proximal tubule was reduced 50% [15]. The vasopressin-regulated water channel aquaporin-2 (AQP_2_) was expressed in both the initial collecting tubule and the cortical collecting duct [16]. The ascending limb of the loop of Henle and distal collecting duct, which are mostly located in the medulla, have lower water permeability. We found significantly lower mean FA values in the renal cortex. The reason for the high medullary anisotropy is probably the radial organization of the tubules and the collecting ducts as they drain into the renal pelvis.

Another finding in our study was the left-right kidney difference in DTI indices. There was a statistically significant difference of cortical FA and λ_1_ values and medullary FA, λ_3_, and RD values between right kidneys and left kidneys. Several previous studies demonstrated small differences between the left and right kidneys. Oh et al. investigated the bilateral renal functional difference by renal scintigraphy using technetium-99m diethylenetriaminepentaacetic acid (^99^mTcDTPA). They found that the left kidneys showed greater function. The average fraction of Ccr of left kidneys was 57.8±10.99 ml/min/1.73 m^2^ compared with right kidneys at 52±11.63 ml/min/1.73 m^2^ [17]. Van Onna et al. investigated the bilateral renal blood flow by ^133^Xenon washout technique. The results showed that asymmetry of RBF was found in 51% subjects [18]. Caralps et al. demonstrated asymmetric alteration of the interlobar and arcuate arteries in 11 of 25 patients. They also reported in 10 of the 11 asymmetry cased that the left kidney was more affected than the right one [19]. Inequality of renal DTI items between the kidneys, therefore, is likely to originate from structural or functional differences, or both.

Our study also showed significant DTI index differences between genders. Women’s medullary λ_2_, λ_3_ and RD values were higher than those values in men. Because the secondary and tertiary eigenvalues λ_2_, λ_3_ and RD reflect transtubular water diffusion motion, we supposed that there was a renal tubule reabsorption difference between male and female subjects. Liu et al. investigated the renal vasopressin V_2_ receptor (V_2_R) in the medullary collecting duct of normal Sprague-Dawley rats. He found that V_2_R mRNA and protein expression was 2.6- and 1.7-fold higher, respectively, in females compared to males [20]. When arginine vasopressin (AVP) interacts with V_2_R, the water permeability of the collecting duct increases through the insertion of AQP_2_ water channels into the apical membranes of renal collecting duct cells [21]. Estrogen can directly interact with the renin-angiotensin-system (RAS) to down-regulate renin and angiotensin converting enzyme 1 (ACE_1_) activity and angiotensin type 1 receptor (AT_1_R) mRNA expression, as well as to up-regulate AT_2_R mRNA expression [22]. Moreover, renal expression of AT_2_R was three-fold greater in female mice compared with males under basal conditions [23]. Studies in genetically engineered mice lacking the AT_2_ receptor have confirmed the concept that the AT_2_ receptor mediates a bradykinin-NO-cGMP vasodilator cascade [24]. This mechanism might expand the diameter of microvascular and could change the secondary and tertiary eigenvalues of DTI.

The effective diffusion tensor is estimated from a series of diffusion weighted images using a relationship between the measured echo attenuation in each voxel and the applied magnetic field gradient sequence. Just as in diffusion imaging where a scalar *b*-factor is calculated for each DWI, in DTI MRI a symmetric *b*-matrix is calculated for each DWI. Whereas the *b*-value summarized the attenuating effect on the MR signal of all diffusion and imaging gradients in one direction, the *b*-matrix summarized the attenuating effect of all gradient waveforms applied in all three direction, *x*, *y* and *z*. DTI MRI is inherently three dimensional; one must apply diffusion gradients along at least six noncollinear, non-coplanar directions in order to provide enough information to estimate the six independent elements [25]. In a study by Thoeny et al, when low *b* values were used for diffusion-weighted imaging, they found no significant difference between the ADC values of the cortex and the medulla in healthy kidney; this result has been attributed to the effect of higher true diffusion in the cortex being counteracted by the greater anisotropy that results from the radial orientation of medulla structures. There was a significant difference among ADC values of the cortex and medulla in a high *b* value group. With high *b* values, the effect of perfusion in cancelled out, and the ADC values reflects mostly diffusion [11].

In many instances, researchers are interested in variable that cannot be directly observed, such as achievement, intelligence, or beliefs. In research methodology, researchers use terms such as latent variables or factors to describe unobserved variables. Factor analysis is statistical techniques that one can use to reduce the number of observed variables into a smaller number of latent variables by examining the covariation among the observed variables. Factor analysis was also used to categorize these parameters and eliminate colinearity. In our study, there were 12 and 7 measurements items of DTI and demographic & anthropometric items, respectively. The correlation analysis showed that there was obvious colinearity in the DTI and demographic & anthropometric items. In the present investigation we used the method of principal-components factors analysis to reduce or rearrange the suet of variables. In DTI parameters, the first and second factor could be interpreted as cortical and medullary water molecular diffusion capability. The third and fourth factor could be interpreted as medullary and cortical water molecular direction capability. In demographic and anthropometric parameters, there were three factors which could be interpreted as physique, gender and aging factors. In factor analysis, a set of variables is reduced into smaller sets of separate dimensions or factors that account for significant portions of the variance in the interrelation among the variables. Four factors of DTI items and three factors of demographic & anthropometric items account for a significant proportion (about 95.86% and 92.50%) of the variance in the interrelationships among a relatively large set of variables. When we clinically evaluated healthy renal water diffusion function, these factors’ equations could provide enough information.

We found a significant positive correlation between aging factor and medullary water molecular direction capability factor. These results implied renal medullary structural and functional changes in aging kidneys. In aging kidneys, tubular dilation and atrophy are typical features of the outer medulla and medullary rays [26]. Renal tubular diverticula and simple renal cysts become increasingly prevalent with age [27], [28]. We speculate that these tubular morphological changes and abnormalities may impact the normal tubular anatomical architecture. Subsequently, medullary FA values measured by DTI-MRI, which reflects direction of water diffusion in the medullary tubules, would also change. Several studies have already demonstrated a decline renal eGFR in aging populations. Rule et al. used nonradiolabeled iothalamate clearance to assess GFR. GFR declined significantly with increasing age. Men aged 20 years had an estimated mean GFR of 129 mL/min. This declined by 4.6 ml/min/decade. Women aged 20 years had a mean GFR of 123 ml/min. A decline of 7.1 ml/min/decade was found [29]. Davies et al. measured GFR by renal inulin clearance and found GFR declined from an average of 123 ml/min per 1.73 m^2^ at the age of 30 years to 65 ml/min per 1.73 m^2^ at the age of 90 years [30]. We found a positive correlation between medullary λ_1_ and eGFR. As the primary eigenvalue λ_1_ reflected water diffusion direction along the tubule, we presumed medullary tubular fluid would decrease in aging kidneys. Several studies have already found that decreased numbers of functional glomeruli might result in hyperfiltration of fluids in the tubular ducts. Hoy et al. estimated the total number of glomeruli by using stereological techniques in autopsied kidneys. Given the increased proportion of sclerosed glomeruli, the total number of functional glomeruli decreased with age [31]. Tan et al. measured the ultrafiltration coefficient (*K_f_*) of the whole kidney and individual glomeruli *K_f_* using light and electron microscopic examination of renal biopsy specimens. The numbers of functional glomeruli appeared to be substantially decreased in older, compared with younger, kidney donors [32]. Collapse of the glomerular tufts accompanies obliteration of preglomerular arterioles. Anatomic shunts between afferent and efferent arterioles are commonly seen in the juxtamedullary region [33].

There were also several limitations in our study. Firstly, the results of DTI measurements might be altered by other medication. Although we excluded most medicines which could impact on renal blood flow or fluid transportation, there were still many other medicines that might influent results of DTI measurement. In a randomized, double-blind, placebo-controlled crossover study, Mose et al. found that atorvastatin increased tubular absorption of sodium and renal nitric oxide (NO). It is well known that NO influences renal hemodynamics and medullary perfusion, since systemic NO inhibition decreased renal plasma flow and medullary capillary blood flow. Statins did not change resting renal plasma flow, but statins increased basal nitric oxide synthase (NOS) activity in renal vasculature. It is possible that statins change the distribution of renal perfusion between the cortical and medullary compartments. Moreover, statin induced change in NO could directly modulate the activity in one or more of the sodium channels in the nephron [34]. Another medicines which could influence renal blood flow are uric acid lowing agents. Previous studies showed that an elevated uric acid has been consistently shown to predict a fall in GFR in the adult without kidney disease. Kanbay et al. found that the eGFR increased 3.3 ml/min/1.73 m^2^ in peoples with allopurinol treatment during 16 weeks [35]. One of the mechanisms is that uric acid may active the RAS by its hemodynamic effects to increase systemic and glomerular pressure. Secondly, the lower diffusion sensitive gradient directions might impact on the accuracy of the DTI results. Unfortunately, we did not explore the different numbers of diffusion sensitive gradient directions in our DTI measurements. As the diffusion tensor has six unique elements, a minimum of six noncollinear diffusion-encoding directions are needed to fully estimate the tensor. The choice of optimal acquisition schemes is a controversial issue regarding the number of diffusion encoding directions (NDED). To increase signal-to-noise ratio (SNR), some studies acquire repeated scans of the same set of diffusion-weighting directions or use more than the minimum six directions to increase the angular resolution of a DTI dataset. Many simulation and experimental studies suggest that the latter is more preferable. One simulation study suggested that at least 20 unique diffusion-encoding directions are required for the robust estimation of anisotropy, and 30 unique diffusion-encoding directions are necessary for the robust estimation of tensor orientation. Liu et al. confirmed that increasing the NDED could improve both the accurate estimation and reproducibility of DTI measurements. Beyond that, acquiring data with more NDED could reduce the difference between inter and intrasession reproducibility, thus promoting the use of DTI measurements in longitudinal studies [36]. Thirdly, the method of selection of ROIs might also influence the DTI results. There is no standard widely accepted method for selection of ROIs and analyzing renal DTI MRI data. In reality, some of the DTI parameters vary gradually from the cortex to the medulla, reaching a most hypoxic zone in the deepest sections of medullary pyramids. Hence, the precision and reproducibility of DTI parameters values are affected by the size and location of the ROI. Larger ROIs that include the entire medullary compartments may provide more representative and less variable mean values, but often include multiple medullary and coticomedullary overlap zones with different hemodynamic. Small, selective ROIs are less vulnerable to volume averaging, but may be skewed by fluctuations caused by spatial and temporal heterogeneity within the kidney, particularly in the medulla.

## Conclusion

DTI-MRI is a novel non-invasive tool used to assess renal fluid diffusion changes. The DTI-MRI technique is useful in evaluating dynamic renal function. Renal water molecular diffusion differences exist in human kidneys and genders. Age and eGFR correlate with some of DTI-MRI indices.Our results provided useful reference data for investigating renal function and clinical diagnosis.
